# Circulating Exosomal miRNA as Diagnostic Biomarkers of Neurodegenerative Diseases

**DOI:** 10.3389/fnmol.2020.00053

**Published:** 2020-04-15

**Authors:** Lin Wang, Lijuan Zhang

**Affiliations:** ^1^Department of Emergency Medicine, Shengjing Hospital of China Medical University, Shenyang, China; ^2^Department of Obstetrics and Gynecology, Shengjing Hospital of China Medical University, Shenyang, China

**Keywords:** biomarker, exosomal miRNA, neurodegenerative disease, CSF, blood

## Abstract

Neurodegenerative diseases (NDDs) are a group of diseases caused by chronic and progressive degeneration of neural tissue. The main pathological manifestations are neuronal degeneration and loss in the brain and/or spinal cord. Common NDDs include Alzheimer disease (AD), Parkinson disease (PD), Huntington disease (HD), and amyotrophic lateral sclerosis (ALS). The complicated pathological characteristics and different clinical manifestations of NDDs result in a lack of sensitive and efficient diagnostic methods. In addition, no sensitive biomarkers are available to monitor the course of NDDs, predict their prognosis, and monitor the therapeutic response. Despite extensive research in recent years, analysis of amyloid β (Aβ) and α-synuclein has failed to effectively improve NDD diagnosis. Although recent studies have indicated circulating miRNAs as promising diagnostic biomarkers of NDDs, the miRNA in the peripheral circulation is susceptible to interference by other components, making circulating miRNA results less consistent. Exosomes are small membrane vesicles with a diameter of approximately 30–100 nm that transport proteins, lipids, mRNA, and miRNA. Because recent studies have shown that exosomes have a double-membrane structure that can resist ribonuclease in the blood, giving exosomal miRNA high stability and making them resistant to degradation, they may become an ideal biomarker of circulating fluids. In this review, we discuss the applicability of circulating exosomal miRNAs as biomarkers, highlight the technical aspects of exosomal miRNA analysis, and review studies that have used circulating exosomal miRNAs as candidate diagnostic biomarkers of NDDs.

## Introduction

Neurodegenerative diseases (NDDs) are a group of diseases caused by chronic and progressive degeneration of neural tissue. The main pathological manifestations are neuronal degeneration and loss in the brain and/or spinal cord (Amin Lari et al., 2019). Common NDDs include Alzheimer disease (AD), Parkinson disease (PD), Huntington disease (HD), and amyotrophic lateral sclerosis (ALS).

Exosomes are extracellular vesicles with a diameter of 30–100 nm. They are produced by a variety of cells in eukaryotes and contain proteins, lipids, mRNA, and miRNAs. Exosomes carry the components of their original cells and interact with adjacent or distant cells to perform information exchange between different cells under both physiological and pathological conditions (Zhang et al., 2015; Yang et al., 2017).

In recent years, studies have shown that misfolded proteins associated with the pathogenesis of NDDs, such as α-synuclein (α-syn), tau, and amyloid β (Aβ), can be transported through exosomes and thereby promote the transmission of these proteins between cells and to nonpathological areas, hastening disease progression (Asai et al., 2015; D’Anca et al., 2019; Hosaka et al., 2019; Jiang et al., 2019). In addition to the ability of misfolded proteins to accelerate the development of NDDs, exosomal miRNAs (ex-miRNAs) are also involved in the pathogenesis of NDDs. AD onset is related to inflammation, which may increase cell damage and cause neuronal death. For example, the levels of ex-let-7 are increased in the brains of AD patients, where it can activate Toll-like receptor and further promote the release of inflammatory factors by activating its downstream signal molecules, eventually leading to neuronal death (Winkler et al., 2014). BACE1 (β-site amyloid precursor protein-cleaving enzyme 1) protein is an endopeptidase that cleaves the β-amyloid precursor protein to generate neurotoxic β-amyloid peptide Aβ_1–42_ (Haniu et al., 2000). Wang et al. (2008) indicated the ex-miR-107 contributes to BACE1 posttranscriptional regulation, which was predicted to exacerbate pathology in AD patients (Wang et al., 2008; Van Giau and An, 2016). Ex-miR-125, ex-miR-210, ex-miR-450b, and ex-miR-669b promote mitochondrial dysfunction, immune system disturbance, and inflammatory activation through multiple signaling pathways to trigger manganese-dependent α-syn overexpression and deposition and thereby play an important role in the pathogenesis of PD (Danzer et al., 2012; Harischandra et al., 2018). Ex-miRNA-7 reduces the expression of α-syn protein, the major component of Lewy bodies in sporadic PD (Junn et al., 2009). Langfelder et al. (2018) suggested that miR-128, miR-132, and miR-218 may be significantly correlated with the CAG repeat expansion of HD. The exosomes of mutant superoxide dismutase 1 (SOD1) motor neurons are rich in ex-miR-124, which can activate microglia and the nuclear factor κB signaling pathway to stimulate the release of numerous cytokines, such as interleukin 1β, tumor necrosis factor α, major histocompatibility class II, and inducible nitric oxide synthase (Pinto et al., 2017). The expression levels of ex-miR-155, ex-miR-146a, and ex-miR-124 are increased in the later stage of ALS, further aggravating the inflammation reaction, leading to a disordered intracellular environment and motor neuron degeneration and necrosis (Pinto et al., 2017). Ex-miR-27a-3p was down-regulated in ALS patients. Xu et al. (2018) suggested the down-regulated ex-miR-27a-3p could mineralize osteoblasts by dysregulating Wnt signaling pathway.

The complicated pathological characteristics and different clinical manifestations of NDDs have led to insufficiently sensitive and efficient diagnostic biomarkers. In addition, no sensitive biomarkers designed to monitor the course of NDDs can predict the prognosis and observe the therapeutic response. Patients with high levels of Aβ42 in the peripheral blood have an increased risk of dementia after 5 years; in addition, peripheral blood levels of tau protein are higher in AD patients and significantly associated with future cognitive impairment (Fiandaca et al., 2015; McDade and Bateman, 2017). However, the relatively low levels of Aβ and tau proteins in peripheral blood necessitate more sensitive detection techniques and increase the detection cost, which limits their application as diagnostic biomarkers of AD. Tabrizi et al. (2013) showed that the plasma levels of neurofilament light chain (NfL) were significantly increased in HD patients and closely associated with age and CAG repeat length. However, the NfL concentration failed to reflect the treatment effects of patients.

Recent studies have suggested that miRNAs in the peripheral circulation are important biomarkers for the evaluation of diseases, including NDDs. In patients with NDDs, specific miRNAs are differentially expressed in body fluids such as blood and interstitial fluids. The plasma levels of miR-206 were determined to be increased in AD and to display a close relationship with cognitive decline and memory deficits (Kenny et al., 2019). Bai et al. (2017) found that decreased levels of serum miR-29, especially miR-29a and miR-29c, were potential biomarkers of PD. Increased levels of miR-100-5p and decreased levels of miR-330-3p and miR-641 were correlated with the total functional capacity of HD patients (Díez-Planelles et al., 2016). The authors therefore suggested that the levels of these circulating miRNAs might be promising biomarkers for monitoring disease progression. De Felice et al. (2014) suggested that miR-338-3p was increased in peripheral leukocytes, serum, and cerebrospinal fluid (CSF) from sporadic ALS patients and considered the miRNA to be a potential biomarker for early diagnosis of sporadic ALS (Díez-Planelles et al., 2016).

MiRNAs are small RNA molecules that are widely studied because of their important posttranscriptional regulatory roles in gene expression in cells and can also be found in exosomes. These ex-miRNAs can be protected by exosomes from degradation by nucleases that are widespread in body fluids. This feature also enables disease diagnosis through the detection of the content of specific miRNAs in exosomes. Because of connecting to the central nervous system (CNS) directly, analysis of CSF can accurately reflect the biochemical changes of the CNS and is an accurate and effective body fluid specimen. However, obtaining CSF is invasive and increases the risk of intracranial infection and is not easily accepted by patients. Ex-miRNAs are also stable in blood and can be reliably detected at low concentrations using today’s sensitive analytical methods; the study of ex-miRNAs in blood is a good sample for noninvasive early diagnosis and prognostic evaluation for NDD patients. In this review, we discuss the applicability of circulating ex-miRNAs as biomarkers, highlight the technical aspects of ex-miR analysis, and review published studies that have used circulating ex-miRNAs as candidate diagnostic biomarkers of NDDs.

## Biological Characteristics of Exosomes

Exosomes are phospholipid bilayer membrane vesicles with a diameter of approximately 30–100 nm. They are rich in protein, lipid, mRNA, and miRNA and are released by membrane fusion into various extracellular and body fluids, such as urine, plasma, breast milk, and CSF. They act locally *via* autocrine and paracrine signaling or enter the blood system and travel to distant cells, directly acting on target cells through receptor ligand binding, endocytosis, and cytoplasmic membrane fusion and participating in complex intercellular material and information exchange (Saman et al., 2012; Kalani et al., 2014; Sarko and McKinney, 2017). Exosomes come from the endosome system. At present, exosome biogenesis is considered to begin with the formation of intraluminal vesicles in the multivesicular body (MVB; Trajkovic et al., 2008).

Exosome formation is roughly divided into three steps: (1) the cell membrane is sunken inward to form intracellular vesicles, namely, early endosomes; (2) early endosomes in turn form multivesicles with multiple vesicles in the cavity by means of endophytic buds to form MVBs; and (3) the MVBs combine with lysosomes: some vesicles within MVBs are degraded by lysosomes, whereas the remaining vesicles fuse with the cell membrane and are released to the outside of the cell in the form of exosomes (Grant and Donaldson, 2009; Shao et al., 2018).

## Exosome Composition and miRNA Synthesis and Packaging

Exosomes are rich in proteins and lipids. According to the Exocarta database^1^, there are more than 8,000 exosome-related proteins and 184 lipids, of which more than 100 proteins are listed as exosomal biomarkers (Keerthikumar et al., 2015). Exosomes are mainly composed of two types of molecules: structural molecules and cargo molecules. Exosomes are released through the endosome pathway, and all contain tetraspanin proteins, Rab GTPases, TSG101, and heat shock proteins (Hsp70 and Hsp90; Stenmark, 2009; Hsu et al., 2010). Cargo molecules are lipids, proteins, and genetic material. Exosomes from different cells carry different proteins. Cells can also release prion-like proteins, such as Aβ, tau, α-syn, and misfolded SOD1, under different pathological and physiological conditions (Arellano-Anaya et al., 2015). The main role of lipid components in exosomes is to regulate exosomal sorting of miRNAs and proteins (Lamichhane et al., 2015). In addition to proteins and lipids, exosomes are also responsible for the transport of genetic material such as DNA, mRNA, miRNA, ribosomal RNA, circular RNA, and long noncoding RNA (Crescitelli et al., 2013). However, miRNA accounts for more than 50% of all exosomal RNA (Huang et al., 2013).

**Figure 1:**
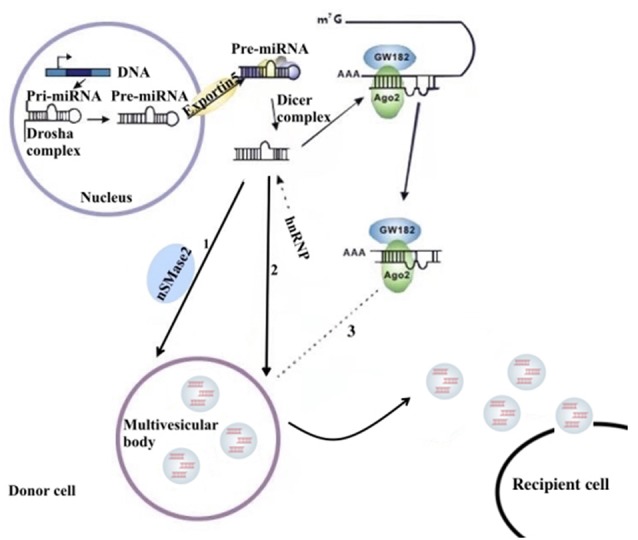
The sorting mechanism for ex-miRNAs. miRNA genes are transcribed into primary miRNAs (pri-miRNAs), and then the precursor miRNAs (pre-miRNAs) are processed by the Drosha complex, which are subsequently delivered into cytoplasm by the Exportin 5 complex. The pre-miRNAs become mature miRNAs digested by the Dicer complex. Mature miRNAs are sorted into exosomes usually by three potential ways: (1) nSMase2 dependent pathway; (2) sumoylated hnRNPs-dependent pathway; (3) miRISC-related pathway. miRISCs colocalize with the sites of exosome biogenesis, and the components, such as Ago2 protein and miRNA-targeted mRNA, are related to sort of miRNAs into exosomes.

miRNAs are a type of endogenous noncoding RNA with regulatory function found in eukaryotes. In the nucleus, the miRNA gene transcribes into the initial miRNA (pri-miRNA) and then the pri-miRNA produces approximately 70 nucleotides with stem-ring structure under the shearing action of Drosha (pre-miRNA). In the cytoplasm, pre-miRNAs are cleaved by the RNase III enzyme Dicer to form a mature double-stranded miRNA containing approximately 21 nucleotides. In the cytoplasm, pre-miRNAs are cleaved by RNA polymerase III to form a mature miRNA containing approximately 21 nucleotides. With the help of helicase, mature miRNAs can form RNA-induced silencing complex (RISC). Under the action of RISC and Argonaute proteins, miRNAs bind to target mRNA. One mature single-stranded miRNA remains in the complex, and the complementary mRNA sites regulate gene expression through base pairing (Higa et al., 2014). miRNAs in the nervous system constitute a complex network for regulating gene expression that plays an important role in the normal physiological regulation of the nervous system and the progression of NDDs (Properzi et al., 2015; Saraiva et al., 2017). The distribution of miRNAs into exosomes in the cytoplasm can be regulated by a variety of mechanisms. Possible pathways include: (1) the neutral sphingomyelinase 2 (nSMase 2)–dependent pathway; (2) the SUMOylated heterogeneous ribonucleoprotein nuclear (hnRNP)–dependent pathways; and (3) the 3′-terminal sequence–dependent pathways of miRNAs (Chevillet et al., 2014; Zhang et al., 2015; Figure 1).

Possible outcomes after exosomes are extracellularly released include: (1) their capture by nearby cells and reabsorption by their secretory cells; (2) their remote relocation, recognition by a cell, and fusion with its cell membrane; and (3) their entrance into the body fluid circulation and movement to other organs; the latter provides a solid foundation for their extraction and analysis. Common fluids for obtaining exosomes of the nervous system are the CSF and peripheral blood (Pant et al., 2012). Studies have found that the analysis of specific ex-miRNAs derived from the nervous system can reflect the physiological condition of the nervous system and provide a reference point for the diagnosis of NDDs.

## Exosome Isolation and Storage

Since the discovery of exosomes, numerous studies have shown that exosomes can play an important role in the diagnosis and treatment of diseases. To facilitate the clinical application of exosomes, efficient separation and storage methods are essential. The current methods for isolating exosomes primarily comprise ultracentrifugation (Baranyai et al., 2015), the microfluidic chip method (Zhang et al., 2019), the antibody affinity capture method (Mathivanan et al., 2010), the polymer precipitation method (Cao F. et al., 2019), and gel exclusion chromatography (Hayashi et al., 2019). Of these, the most commonly used method is ultracentrifugation. The separation principle is based on particle size and density and involves low- and high-speed centrifugation. The method is simple and suitable for large-volume samples, but the equipment cost is high, and the recovery rate unstable. At the same time, the disadvantages of repeated centrifugation, which may damage the vesicle membrane, limit its application (Helwa et al., 2017). The microfluidic chip method can construct a three-dimensional microenvironment based on exosome specificity and separate exosomes from the complex cell matrix. This method can obtain highly pure exosomes with high separation efficiency and automatic control. It is also an ideal method to separate exosomes (Cao H. et al., 2019). The antibody affinity capture method uses the antigens present in the exosomes and the highly specific affinity between the antibodies for separation. This method can also obtain high-purity exosomes and isolate different subtypes of exosomes (Popovic et al., 2018). The polymer precipitation method produces more heteroproteins and may yield particles of uneven size (Niu et al., 2017), whereas gel exclusion chromatography requires special equipment and has a long running time (Monguió-Tortajada et al., 2019), which is why these two methods are not widely used.

In addition, in terms of storage, it is extremely important to maintain the *in vitro* integrity of the exosomes. Exosomes prevent RNase degradation and are considered to be a stable source of miRNA (Koga et al., 2011). Indeed, ex-miRNAs have been shown to be stable for 5 years at −20°C, unaffected for 2 weeks at 4°C, and resistant to freeze–thaw cycles (Weber et al., 2010). Therefore, exosomes, as a source of miRNAs, are efficient means for the storage and recovery of miRNAs under conditions that normally degrade free miRNAs (Thind and Wilson, 2016). Because of their availability and stability, ex-miRNAs are considered to be a new, noninvasive diagnostic biomarker of disease with potential for predicting prognosis. Two miRNA measurement techniques commonly used in research today are microarrays and reverse transcription–polymerase chain reaction (RT-PCR). Microarrays can provide the entire genome expression profiles of miRNAs, which can help to detect a large number of abnormal miRNAs. Compared with RT-PCR, microarrays have higher specificity but lower sensitivity (Mestdagh et al., 2014). Reverse transcription–PCR is more sensitive and suitable for monitoring low-level miRNAs. However, miRNAs have “isomiRs” (sequence heterogeneity at the 3′ and 5′ ends), which may complicate the measurement, especially for RT-PCR (Lee et al., 2010).

Next-generation sequencing, a recently adopted and powerful method for measuring miRNA expression profiles, may prove clinically useful. This technique can accurately quantify miRNAs across the genome and distinguish between miRNAs that differ by only one nucleotide. Because no primers or probes are needed, it can detect new miRNAs (Liguori et al., 2018; Zhou et al., 2018).

**Figure 2:**
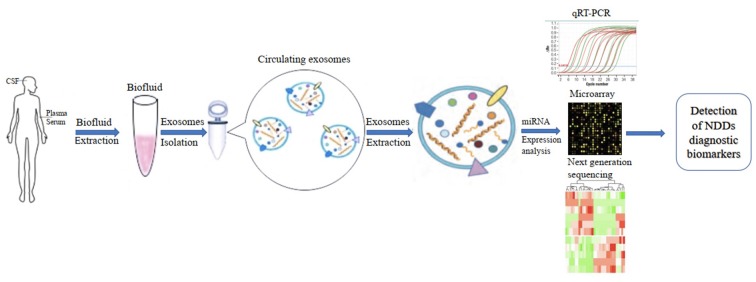
Applications of circulating exosomal miRNA as diagnostic biomarkers of neurodegenerative diseases (NDDs).

In addition, the development of diagnostic biomarkers of NDDs based on circulating ex-miRNAs requires the consideration of multiple factors, such as source fluids, separation techniques, and quantitative methods. These factors should also be considered when published studies in this area are being compared (Figure 2).

## Circulating Ex-miRNAs as Biomarkers in NDDs

**Figure 3:**
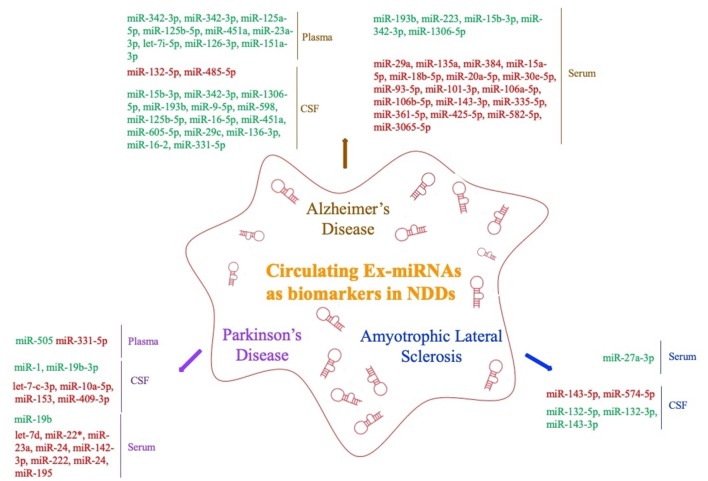
Circulating exosomal miRNAs as biomarkers in NDDs (red fonts represent up-regulated ex-miRNAs; green fonts represent down-regulated ex-miRNAs).

The levels of miRNAs in peripheral blood are affected by multiple factors. In addition to sex, ethnicity, inflammatory factors, and lifestyle (Fan et al., 2015; Wagner et al., 2016; Pheiffer et al., 2018; Ludwig et al., 2019), they may also vary among sample types (whole blood, serum, and plasma; Blondal et al., 2013). Ex-miRNAs effectively avoid the above problems due to their stable expression, and the development and application of high-throughput sequencing technology to miRNA expression profiles have greatly improved diagnostic sensitivity (Cheng et al., 2013; Chen et al., 2017). The pathological changes in the nervous system microenvironment will affect the type and content of exosomes released by nerve cells, and miRNAs, as important components of neuroepigenetics, are often significantly altered as well, which suggests that ex-miRNAs are of considerable value as early diagnostic markers of NDDs (Table 1, Figure 3).

**Table 1:** Circulating ex-miRNAs as biomarkers in neurodegenerative diseases (NDDs).

NDDs	Sample	Sample size	Validated changes		ROC curve analysis	References
			Up-regulated	Down-regulated		
AD	Plasma	46 P, 41 C		ex-miR-342-3p	N	Lugli et al. (2015)
	Plasma	97 P, 97 C		ex-miR-342-3p, ex-miR-125a-5p, ex-miR-125b-5p and ex-miR-451a	N	Rani et al. (2017)
	Plasma	10 P, 15 C		ex-miR-23a-3p, ex-let-7i-5p, ex-miR-126-3p and ex-miR-151a-3p	Y	Gámez-Valero et al. (2019)
	Serum	30 P, 30 C	ex-miR-29a		Y	Barbagallo et al. (2019)
	Serum	208 P, 228 C	ex-miR-135a, ex-miR-384	ex-miR-193b	Y	Yang et al. (2018)
	Serum	16 P, 22 C		ex-miR-223	Y	Wei et al. (2018)
	Serum	23 P, 23 C	ex-miR-15a-5p, ex-miR-18b-5p, ex-miR-20a-5p, ex-miR-30e-5p, ex-miR-93-5p, ex-miR-101-3p, ex-miR-106a-5p, ex-miR-106b-5p, ex-miR-143-3p, ex-miR-335-5p, ex-miR-361-5p, ex-miR-425-5p, ex-miR-582-5p, ex-miR-3065-5p	ex-miR-15b-3p, ex-miR-342-3p, ex-miR-1306-5p	N	Cheng et al. (2015)
	CSF	51 P, 84 C		ex-miR-193b	N	Liu et al. (2014)
	CSF	10 P, 10 C		ex-miR-9-5p, ex-miR-598	Y	Riancho et al. (2017)
	CSF	17 P, 12 C		ex-miR-125b-5p was increased, ex-miR-16-5p, ex-miR-451a, ex-miR-605-5p	Y	McKeever et al. (2018)
	CSF	27 P, 28 C	ex-miR-132-5p, ex-miR-485-5p	ex-miR-29c, ex-miR-136-3p, ex-miR-16-2, ex-miR-331-5p	Y	Gui et al. (2015)
PD	Plasma	52 P, 48 C	ex-miR-331-5p	ex-miR-505	Y	Yao et al. (2018)
	Serum	30 P, 30 C	ex-let-7d, ex-miR-22*, ex-miR-23a, ex-miR-24, ex-miR-142-3p, and ex-miR-222		Y	Barbagallo et al. (2019)
	Serum	109 P, 43 C	ex-miR-24, ex-miR-195	ex-miR-19b	Y	Cao et al. (2017)
	CSF	47 P, 27 C	ex-let-7-c-3p, ex-miR-10a-5p, ex-miR-153, and ex-miR-409-3p	ex-miR-1, ex-miR-19b-3p	N	Gui et al. (2015)
ALS	Serum	10 P, 20 C		ex-miR-27a-3p	N	Xu et al. (2018)
	CSF	22 P, 24 C	ex-miR-143-5p, ex-miR-574-5p	ex-miR-132-5p, ex-miR-132-3p, ex-miR-143-3p	N	Freischmidt et al. (2013)

*CSF, Cerebrospinal fluid; P, patients; C, Controls; ROC, receiver operating characteristic; Y, yes; N, no*.

## Ex-miRNAs as Biomarkers in AD

Lugli et al. (2015) successfully isolated exosomes from the plasma of AD patients and used high-throughput sequencing technology to compare their miRNA expression levels with those of a control group. They found that ex-miR-342-3p levels were significantly lower in AD patients and were highly correlated with several other low-expressing miRNAs. Decreased plasma levels of ex-miR-342-3p were also observed in AD patients by Rani et al. (2017); in addition, ex-miR-125a-5p, ex-miR-125b-5p, and ex-miR-451a levels were also lower in AD patients, and the levels of these ex-miRNA reductions correlated with the extent of cognitive impairment, which was assessed by Montreal Cognitive Assessment scores. Another plasma-based ex-miRNA study by Gámez-Valero et al. (2019) included 10 AD patients and 15 healthy controls. They used next-generation sequencing technology to identify significantly decreased levels of ex-miR-23a-3p, ex-let-7i-5p, ex-miR-126-3p, and ex-miR-151a-3p in AD patients, suggesting that the changes in the plasma levels of ex-miRNAs exhibited diagnostic value for AD.

Barbagallo et al. (2019), who compared the levels of ex-miRNAs in the serum of healthy controls and patients with different types of NDDs, found that ex-miR-29a levels were significantly increased in AD patients [area under the curve (AUC), 0.71; 95% confidence interval (CI), 0.577–0.843], with 43.0% sensitivity and 97.0% specificity and a stronger fold change and higher *p* value than free miR-29a in serum. Yang et al. (2018) used quantitative RT (qRT)-PCR to measure the serum levels of three ex-miRNAs—ex-miR-135a, ex-miR-193b, and ex-miR-384 in 101 mild cognitive impairment (MCI) and 107 dementia of Alzheimer type (DAT) patients. They found with high sensitivity and specificity that the levels of ex-miR-135a (AUC, 0.981; 95% CI, 0.951–0.995) and ex-miR-384 (AUC, 0.870; 95% CI, 0.816–0.914) were increased in DAT patients and that ex-miR-193b (AUC, 0.798; 95% CI, 0.736–0.914) was decreased. Moreover, they further determined that the combination of the three miRNAs would be better for the early diagnosis of AD than any single one. The serum levels of ex-miR-223 were also found to be dysregulated in AD patients, with Wei et al. (2018) suggesting that the level of ex-miR-223 (AUC, 0.875; 95% CI, 0.7779–0.9721) was significantly decreased in AD patients and might act as a biomarker to distinguish AD patients from individuals without dementia. Cheng et al. (2015) explored serum-derived differentially expressed ex-miRNAs in a study of AD patients and identified 14 significantly upregulated ex-miRNAs (ex-miR-15a-5p, ex-miR-18b-5p, ex-miR-20a-5p, ex-miR-30e-5p, ex-miR-93–5p, ex-miR-101–3p, ex-miR-106a-5p, ex-miR-106b-5p, ex-miR-143–3p, ex-miR-335-5p, ex-miR-361-5p, ex-miR-425-5p, ex-miR-582-5p, and ex-miR-3065-5p) and 3 significantly downregulated ex-miRNAs (ex-miR-15b-3p, ex-miR-342-3p, and ex-miR-1306-5p). Although all ex-miRNAs were validated by qRT-PCR analysis, their study lacked receiver operating characteristic (ROC) curve analysis and enrolled fewer patients with MCI. Nonetheless, their results contribute to the study of serum-derived ex-miRNAs as markers for AD diagnosis.

By comparing ex-miR-193b levels between the normal population and patients with MCI and DAT, Liu et al. (2014) found that ex-miR-193b levels were significantly lower in patients with MCI and DAT than in the people in the control group. However, the difference in the levels of miR-193b in whole blood was not statistically significant. In addition, ex-miR-193b levels were lower in DAT patients than in MCI patients. This study suggested that specific ex-miRNAs can be used as diagnostic biomarkers to reflect AD disease progression. Another study that examined the levels of free miRNAs and ex-miRNAs in the CSF of AD patients found that ex-miR-9-5p and ex-miR-598 levels were significantly downregulated. However, free miR-9-5p and miR-598 were detected in up to 50% and 75% of healthy control CSF samples, respectively, but they were not present in any AD CSF sample. This difference suggested that ex-miRNAs may be more reliable as potential biomarkers of AD (Riancho et al., 2017).

Researchers have also strived to distinguish between young- and late-onset AD (YOAD/LOAD). McKeever et al. (2018) collected CSF samples from 17 YOAD patients, 13 LOAD patients, and 12 healthy controls; they found that CSF-derived ex-miR-125b-5p was increased in YOAD patients, whereas ex-miR-16-5p, ex-miR-451a, and ex-miR-605-5p were decreased. In addition, the altered levels of ex-miR-125b-5p, ex-miR-451a, and ex-miR-605-5p were similar in both LOAD and YOAD patients when compared with the healthy controls. Another study reported decreased levels of ex-miR-29c, ex-miR-136-3p, ex-miR-16-2, and ex-miR-331-5p but increased levels of ex-miR-132-5p and ex-miR-485-5p in the CSF of AD patients (Gui et al., 2015).

## Ex-miRNAs as Biomarkers in PD

PD is the second most common NDDs in the elderly. The incidence of PD increases with age. Compared with the direct detection of biomarkers such as DJ-1, oxDJ-1, α-syn, and miRNA in the CSF or blood, exosome detection is more stable and more reliable and can better reflect PD severity (Hartfield et al., 2012; Saito, 2017). Cerebrospinal fluid and plasma exosomes are rich in miRNA and provide a stable protective environment for genetic material. The expression levels of miRNA are significantly altered in PD, indicating that these nucleic acids play a major role in the pathogenesis of PD and may become biological markers of PD.

Yao et al. (2018) compared the diagnostic value of plasma-derived ex-miRNAs in patients with PD. Their study included 52 PD patients and 48 healthy controls. The analysis was verified by qRT-PCR and showed that the ex-miR-331-5p expression level was significantly increased and the ex-miR-505 expression level significantly decreased in PD patients, with AUCs of 0.849 and 0.898, respectively, suggesting that these two ex-miRNAs are potentially useful for the early diagnosis of PD.

Barbagallo et al. (2019) isolated ex-miRNA from the serum of 30 PD patients and compared it with that of 30 healthy controls to try to explore the differential expression of ex-miRNAs in PD patients. The results were verified by TaqMan RT-PCR analysis. The expression levels of ex-let-7d, ex-miR-22* (asterisk indicates anti-sense miR), ex-miR-23a, ex-miR-24, ex-miR-142-3p, and ex-miR-222 were significantly increased in the serum of PD patients. In addition, ROC curve analysis revealed that these six ex-miRNAs are ideal biomarkers for the diagnosis of PD, but the researchers also mentioned that, because of the small number of patients enrolled, subsequent larger multicenter studies were needed to verify their conclusions. Another differential expression study of serum-derived ex-miRNA was performed by Cao et al. (2017). Comparison of the levels of 24 ex-miRNAs from the serum of 109 PD patients and healthy controls showed that the levels of ex-miR-24 (AUC, 0.908; 95% CI, 0.850–0.949) and ex-miR-195 (AUC, 0.697; 95% CI, 0.617–0.770) were increased, and ex-miR-19b (AUC, 0.753; 95% CI, 0.675–0.820) decreased in PD patients, indicating the possible use of ex-miRNA as a novel strategy for the diagnosis of PD.

Gui et al. (2015) performed the only differential expression analysis of ex-miRNA derived from the CSF of PD patients. The study included 47 PD patients, 28 AD patients, and 27 healthy controls. The researchers verified 16 differentially expressed ex-miRNAs from 746 miRNAs. Among the 11 downregulated ex-miRNAs, the levels of ex-miR-1 and ex-miR-19b-3p were significantly decreased in the CSF of PD patients, whereas the levels of ex-let-7-c-3p, ex-miR-10a-5p, ex-miR-153, and ex-miR-409-3p were significantly increased. The researchers further compared the expression levels of ex-miRNAs in the CSF of AD patients, with the results showing that the expression levels of ex-let-7-c-3p, ex-miR-10a-5p, ex-miR-153, and ex-miR-409-3p were significantly higher, and the levels of ex-miR-1 and ex-miR-19b-3p significantly lower, in the CSF of PD patients than those of AD patients. The final results of the study suggested that the expression of CSF-derived ex-miRNAs in PD patients not only had potential diagnostic value for PD, but also could help to identify different types of NDDs.

## Ex-miRNAs as Biomarkers in HD

HD is a hereditary and slowly progressing NDD. Diagnosis mainly relies on family genetic history and genetic testing. Although HD is an untreatable disease, biomarkers that might provide early diagnostic clues or reflect disease progression are still important for patients. Progress has been made in circulating biomarkers of diagnosis of HD, with plasma NfL reported to be significantly increased in HD patients (Tabrizi et al., 2013). Leukocyte telomere length values were significantly decreased in HD and might be a reliable biomarker to track HD progression (Scarabino et al., 2019). In the PREDICT-HD study, the researchers suggested that six miRNAs—miR-135b-3p, miR-140-5p, miR-520f-3p, miR-3928-5p, miR-4317, and miR-8082-were significantly increased in presymptomatic HD patients and could be potential biomarkers for the early diagnosis of HD (Reed et al., 2018). Gaughwin et al. (2011) found that the level of plasma miR-34b was significantly decreased in presymptomatic HD patients compared with healthy controls, suggesting miR-34b as a new potential biomarker of HD that can be stably expressed in plasma and detected before clinical symptoms occur. No study has explored ex-miRNAs as biomarkers for the diagnosis of HD. However, previous studies have found that miR-124 expression decreases in HD patients and can lead to upregulation of REST expression, thereby inhibiting the expression of brain-derived neurotrophic factor and indicating that abnormal expression of miR-124 plays a key role in the pathogenesis of HD (Cheng et al., 2009; Das et al., 2013; Hwang and Zukin, 2018).

Lee et al. (2017) developed an exosome-based delivery method (Exo-124) to treat HD in an animal model (R6/2 transgenic HD mice). Although they did not achieve the desired improvement in motor symptoms, their research into a new exosome-based system for miRNA delivery in NDDs is nonetheless valuable.

## Ex-miRNAs as Biomarkers in ALS

ALS is one of the NDDs entailing selective upper and lower motor neuron damage. The main clinical manifestations of ALS are rapidly progressing muscle weakness and atrophy and ultimately death due to respiratory failure caused by respiratory muscle weakness. The early symptoms of ALS are not specific, and it can be easily confused with various diseases such as cervical spondylosis and myasthenia gravis. It lacks specific biomarkers, which makes clinical diagnosis difficult, and has a high rate of misdiagnosis (Worms, 2001). Because the molecules contained in exosomes are specific and related to their source cells and pathological conditions, exosomes can reflect the physiological and pathological changes of the original cells, including proteins, mRNA, and miRNA, and have potential biomarker functions (Bang and Thum, 2012). The serum levels of miR-1234-3p and miR-1825 have been reported to be significantly decreased in ALS patients, with the miR-1825 decrease observed in both sporadic ALS and familial ALS patients and the miR-1234-3p decrease restricted to patients with sporadic ALS (Freischmidt et al., 2015). The plasma levels of miR-130a-3p, miR-151b, and miR-221-3p were also reported to be decreased in sporadic ALS patients and positively correlated with sporadic ALS progression, suggesting that these miRNAs could be useful not only as biomarkers for diagnosis, but also for monitoring disease progression (Liguori et al., 2018).

A study of serum-derived ex-miRNA as a biomarker for ALS diagnosis performed by Xu et al. (2018) enrolled 10 ALS patients and 20 healthy controls. By comparing the two groups, they found that ex-miR-27a-3p levels were significantly decreased in ALS patients. The researchers concluded that ex-miR-27a-3p might be a potential diagnostic biomarker of ALS. However, because the study did not further analyze the data using ROC statistics, specific analysis is needed before this test can be applied in the clinic. Freischmidt et al. (2013) determined the expression levels of ex-miRNAs in the CSF and serum of 22 patients with sporadic ALS and compared them with those of 24 healthy controls. Ex-miR-132-5p, ex-miR-132-3p, and ex-miR-143-3p were significantly decreased, and ex-miR-143-5p and ex-miR-574-5p significantly increased in ALS patients, suggesting that these ex-miRNAs are potential biomarkers for ALS diagnosis. In addition, all of these ex-miRNAs can be combined with TDP-43 *in vitro*, proving that miRNA dysfunction may participate in ALS pathogenesis by affecting TDP-43.

## Advantages and Disadvantages of Ex-miRNA as Diagnostic Biomarkers for NDDs

The stable expression of ex-miRNAs and their resistance to the influence of external factors that might affect their expression, coupled with the development and application of high-throughput sequencing technology for determining the expression profiles of miRNAs, greatly improve the sensitivity of ex-miRNA diagnosis (Cheng et al., 2013). Some studies have compared ex-miRNAs with currently recognized methods for diagnosing NDDs to further illustrate their accuracy as NDD biomarkers. Cheng et al. (2015) analyzed the plasma ex-miRNAs of AD patients and identified a group of abnormally expressed ex-miRNAs. Compared with other diagnostic methods such as neuropsychology and neuroimaging, the sensitivity and specificity of ex-miRNAs were 87% and 77%, respectively, which showed that the specific ex-miRNAs had potential value as biomarkers for AD diagnosis. Liu et al. (2014) examined miR-193b levels in the peripheral blood of healthy controls, patients with MCI, and patients with DAT. By comparing ex-miR-193b, Aβ, tau, p-tau, HCY, homocysteine; APOE, apolipoprotein E levels, the authors found that ex-miR-193b levels were significantly lower in MCI and DAT patients than in healthy controls; there were no significant differences in peripheral blood. In addition, ex-miR-193b expression was lower in DAT patients than in MCI patients, and ex-miR-193b was negatively correlated with Aβ42. This study indicated that specific ex-miRNAs may have similar diagnostic efficacy to traditional biomarkers and could be used as potential diagnostic biomarkers to reflect AD disease progression. Thus, it can be seen that ex-miRNA have considerable potential as biomarkers for the diagnosis and prognosis prediction of NDDs.

In the field of NDD diagnosis, analysis of circulating ex-miRNAs is undoubtedly a novel and particularly exciting method. However, given the relatively late clinical start for exosomal research, it still faces significant practical problems. First, existing exosomal enrichment schemes differ in the yield of exosomal isolation and may also result in slightly different amounts of exosomes (Taylor et al., 2011; Rekker et al., 2014). These methods include ultracentrifugation, density gradient separation, immunoaffinity capture, size-exclusion chromatography, and commercial kits (Zhang et al., 2015). Depending on the sample tested, they may all isolate only small amounts of exosomes. In these cases, a large number of test samples may be required. However, a study has shown that as little as 250 μl of human plasma might be sufficient for the isolation of an adequate quantity of ex-miRNAs (Huang et al., 2013). Some commercial reagents can simplify the process of exosomal extraction, but they are often too expensive to be widely used in clinical diagnosis. Second, there is no effective and rapid technical method to detect exosomes. Moreover, the method for long-term *in vitro* storage of exosomes requires optimization. Third, there is huge variation in the ex-miRNA expression results among different epidemiological studies of NDD patients in the progressive stage, indicating the need for a large sample of standardized and controllable epidemiological research data to determine the diagnostic efficacy.

However, for ex-miRNAs to become the diagnostic biomarker for NDDs, breakthroughs need to be made in the following aspects: (1) clarification of the relationship between ex-miRNAs and the occurrence and development of NDDs and further elucidation of disease pathogenesis; (2) development of more mature and reliable techniques for extracting ex-miRNAs from various body fluids for their application in clinical practice; and (3) using ex-miRNAs as a breakthrough point, identification of more specific miRNAs in various NDDs as biomarkers for early diagnosis, thereby boosting clinical diagnosis.

## Conclusions

Although still facing significant practical problems, the stable expression of ex-miRNAs has been a potential biomarker of NDDs. As a possible diagnostic biomarker of NDDs, ex-miRNAs cannot currently surpass the traditional clinical detection methods, but with technological and research development, their advantages as diagnostic biomarkers of NDDs will undoubtedly be gradually validated, providing a theoretical basis for the early detection and prevention of NDDs.

## Author Contributions

This manuscript was primarily written by LW. The figure was produced by LW and LZ. LZ contributed to the editing of this review. Both authors read and approved the final manuscript.

## Conflict of Interest

The authors declare that the research was conducted in the absence of any commercial or financial relationships that could be construed as a potential conflict of interest.

## Footnotes

^1^http://www.exocarta.org
